# Digital assessment of nonverbal behaviors forecasts first onset of depression

**DOI:** 10.1017/S0033291724002010

**Published:** 2024-09

**Authors:** Sekine Ozturk, Scott Feltman, Daniel N. Klein, Roman Kotov, Aprajita Mohanty

**Affiliations:** 1 Department of Psychology, Stony Brook University, Stony Brook, NY, USA; 2 Department of Applied Mathematics and Statistics, Stony Brook University, Stony Brook, NY, USA; 3 Department of Psychiatry and Behavioral Science, Stony Brook University, Stony Brook, NY, USA

**Keywords:** depression, early identification, FaceReader, facial recognition, nonverbal behaviors

## Abstract

**Background:**

Adolescence is marked by a sharp increase in the incidence of depression, especially in females. Identification of risk for depressive disorders (DD) in this key developmental stage can help prevention efforts, mitigating the clinical and public burden of DD. While frequently used in diagnosis, nonverbal behaviors are relatively understudied as risk markers for DD. Digital technology, such as facial recognition, may provide objective, fast, efficient, and cost-effective means of measuring nonverbal behavior.

**Method:**

Here, we analyzed video-recorded clinical interviews of 359 never-depressed adolescents females via commercially available facial emotion recognition software.

**Results:**

We found that average head and facial movements forecast future first onset of depression (AUC = 0.70) beyond the effects of other established self-report and physiological markers of DD risk.

**Conclusions:**

Overall, these findings suggest that digital assessment of nonverbal behaviors may provide a promising risk marker for DD, which could aid in early identification and intervention efforts.

Depressive disorders (DD) are a debilitating public health problem and one of the leading causes of disability worldwide (Kessler & Bromet, 2013). The incidence of DD rises dramatically in adolescence (Birmaher et al., 2007), with an especially marked increase in females who are at two times higher risk for experiencing depression compared to their male counterparts (Salk, Hyde, & Abramson, 2017). Therefore, adolescence, particularly for females, is an important developmental stage to identify objective, cost-effective and non-invasive markers of risk and resilience, allowing for better prevention and treatment of DD (Leventhal, Pettit, & Lewinsohn, 2008; Mittal & Wakschlag, 2017).

In addition to evaluations of self-reported symptoms, clinical scientists have long been studying biological markers of vulnerability to DD using methods such as neuroimaging and genetics; however, the complexity and high costs of these methods limit their use for diagnostic and preventative purposes in the real world. Utilization of artificial intelligence (AI) and digital technology measuring client's verbal and non-verbal behavior in clinical settings offers significant promise (Cohn et al., 2018; Eichstaedt et al., 2018), due to the objective, efficient, and cost-effective nature of these techniques. In this domain, non-verbal signs of DD become a particular opportunity for digital technology. Clinicians have long relied on non-verbal signs such as psychomotor retardation or agitation and affective expressions (Fairbanks, McGuire, & Harris, 1982) in evaluating risk and making diagnostic decisions regarding depression; however, these methods lack reliability (Lanyon & Wershba, 2013; Levin-Aspenson & Watson, 2018; Snowden, 2003). Digital phenotyping or the use of digital technology to comprehensively measure behavior, has been shown to quantify nonverbal signs of DD (Abbas et al., 2021a) and is postulated to tap into the underlying biological phenotypes related to the clinical dysfunction (Insel, 2017; Abbas, Schultebraucks, & Galatzer-Levy, 2021b). These methods have been demonstrated to successfully distinguish nonverbal behaviors, such as head movements and facial expressions, of clinically depressed individuals from those of control participants (Abbas et al., 2021a, 2021c; Pampouchidou et al., 2020), however the power of these digital measures in forecasting depression from adolescence remains unknown.

Nonverbal behaviors encompass a range of observable actions, such as gross motor activity, posture, movements of head, hands, torso and limbs, facial mobility, and eye glances (Friedman et al., 1974; Sobin, 1997). Numerous studies have highlighted distinctions in nonverbal behaviors between depressed individuals and other groups (non-clinical and other clinically diagnosed). These distinctions encompass a variety of nonverbal behaviors, including gross motor behaviors such as psychomotor retardation or agitation, as well as finer facial movements (Kring & Stuart, 2008; Sobin, 1997; Woody et al., 2019).

Early electromyography (EMG) studies demonstrate that depression is linked with reduced facial muscle activity (Gehricke & Shapiro, 2000; Greden, Genero, Price, Feinberg, & Levine, 1986). Particularly, there is diminished movement in zygomatic major muscles producing smiles (Chentsova-Dutton, Tsai, & Gotlib, 2010; Gaebel & Wölwer, 2004; Trémeau et al., 2005) and increased tension in the corrugator muscle that lowers the eyebrows and contributes to frowning facial expressions (Greden, Genero, & Price, 1985; Schwartz et al., 1978). These changes in EMG activity are shown to predict treatment outcome (Greden, Price, Genero, Feinberg, & Levine, 1984). However, a drawback lies in their limited ecological validity due to the fact that this data is obtained in highly controlled settings.

In contrast, employing digital tools for nonverbal behavior analysis offers advantages in naturalistic settings. Recent research utilizing automatic facial movement detection through digital methods revealed that individuals with higher levels of depression exhibit distinct differences in facial expressions. These differences include reduced smiling, more frequent displays of contempt and embarrassment (Girard et al., 2014), decreased synchrony in facial expressions (Altmann, Brümmel, Meier, & Strauss, 2021), and less intense movements around the mouth and eyelids (Stolicyn, Steele, & Seriès, 2022). Additionally, models using the Facial Action Coding System (FACS; Ekman, Friesen, & Hager, 2002) have achieved 79% accuracy in automatically detecting depression through facial movements (Cohn et al., 2009). Therefore, preliminary evidence suggests that automatic detection of facial expressions using digital technology may offer insights into predicting the clinical course and severity of depression (Dibeklioğlu, Hammal, & Cohn, 2018; Gavrilescu & Vizireanu, 2019; Kacem, Hammal, Daoudi, & Cohn, 2018).

Research on digital phenotyping of nonverbal behavior in depression extends beyond facial expressions. Previous studies indicate that depression is associated with diminished change in head position and slower movements (Alghowinem, Goecke, Wagner, Parkerx, & Breakspear, 2013; Joshi, Goecke, Parker, & Breakspear, 2013). AI models predicted depression up to 65% with head motion alone, and up to 78–85% when combined with other nonverbal behaviors including facial movements (Dibeklioğlu et al., 2018), or with acoustic features. This relationship was shown to be dose-dependent such that the movement characteristics were related to the severity of illness as well as treatment progress and remission (Girard et al., 2014; Kacem et al., 2018). Notably, a smartphone-based video analysis by Abbas et al. demonstrated that positive response to treatment was linked with an increase in head movements in depressed participants (2021a). While consistently identifying depression in presently depressed adults, the research exhibits limitations, primarily relying on cross-sectional designs, with the exception of only a handful of studies delving into treatment response, and constrained by small sample sizes.

Rates of depression increase dramatically in adolescence, and the female preponderance emerges around this age, therefore it is critical to identify markers of risk in this period for targeted intervention efforts. Based on the current literature on adults, it is unknown if the behavioral abnormalities captured by facial recognition are present in adolescents. Furthermore, it is unknown whether these are risk factors or correlates of depression, as prior digital assessment studies investigated nonverbal behaviors in depressed individuals. The current evidence has not explored whether these behavioral abnormalities are limited to a depressed state, or if digital tools can reveal risk markers that are evident even before the onset of depression. Most important of all, with the present evidence, it is unclear whether digital tools have a clinical utility that is as good as, or better than established clinical, self-report, and physiological indices of risk for depression. It is of utmost importance to compare digital methods to established predictors of depression in order to demonstrate their incremental validity beyond what is currently available. The present study aims to fill these significant gaps in the literature by examining digitally assessed nonverbal behaviors during a clinical interview in a large community sample of adolescents up to 3 years prior to the onset of depression. In this longitudinal study, never-depressed, healthy adolescent females were followed for 3 years and were evaluated for a wide range of depression risk factors including self- and parent-reported mood and personality measures, family history and physiological variables. Thereby, the present study has two aims. First, it seeks to investigate whether digitally assessed nonverbal behaviors during a clinical interview, measured by a state of the art commercially available software (FaceReader; Noldus Information Technology, 2010), can forecast depression longitudinally over 3 years in a community sample of adolescent females. Specifically, here we focus on head motions and fine facial movements, quantified by FACS, implementing a data-driven approach. We hypothesize that, consistent with previous research, reduced average facial and head movements will predict a DD at 3-year follow-up. Second, we aim to demonstrate whether digitally assessed nonverbal behaviors have incremental validity along with better established predictors of future onset depression. Based on the prediction accuracy reported in the previous literature, we hypothesize that nonverbal behaviors will demonstrate incremental validity.

## Method

### Participants

Participants were recruited from the Suffolk County area in New York, USA as part of the longitudinal Adolescent Development of Emotions and Personality Traits (ADEPT) study which aims to examine factors predicting the onset of DD in adolescent females. At the initial enrollment, a total of 550 adolescent females participated in the study. Both adolescents and their parents provided written informed consent prior to participation and the study was approved by Stony Brook University's Institutional Review Board. Exclusion criteria in the study included history of Diagnostic and Statistical Manual (DSM-IV) Major Depressive Disorder (MDD) or Dysthymia before baseline, an intellectual disability, absence of a participating biological parent, inability to read and/or comprehend questionnaires, and a lack of proficiency in English. As part of the larger study, participants were followed at multiple time points throughout the following 3 years. For the purposes of the current study, we have used baseline and the 3-year follow-up data in the analyses. Thirty five participants were excluded due to presence of a DD not otherwise specified (DD-NOS) before baseline, 120 participants were excluded due to missing analyzable video data from the baseline assessment and 36 participants were excluded due to missing diagnostic data at the 3-year follow-up, resulting in a final sample of 359 adolescents (mean age = 14.38, s.d. = .63, range = 13–15 years, 90.3% Caucasian, 65.5% with at least one college educated parent; see online Supplementary Table S1 for further demographic breakdown).

### Measures

#### Diagnostic assessment

DSM-IV diagnoses were assessed at baseline and 3-year follow-up, using the semi-structured diagnostic interview Kiddie Schedule for Affective Disorders and Schizophrenia for School Aged Children, Present and Lifetime Version (KSADS-PL) (Kaufman et al., 1997). Extensively trained research personnel, overseen by clinical psychologists, conducted the interviews. The KSADS-PL has excellent reliability and validity in diagnosing adolescent psychopathology (Kaufman et al., 1997). Diagnostic status at the 3-year follow-up was a dichotomous variable indicating presence or absence of any DD, which included DSM-IV diagnoses of major depressive episode (MDE), dysthymic disorder, and DD-NOS. We operationalized DD-NOS as a clinically significant depressive episode, characterized by presence of depressed mood, loss of interest or pleasure, or suicidality and clinically significant impairment or treatment that did not meet the full criteria for MDE or dysthymic disorder. The inter-rater reliability for any DD diagnosis across study time points was high based on 48 audio-recorded interviews (kappa = 0.81; Michelini et al., 2021). Participants were video recorded while completing the baseline KSADS-PL diagnostic interview. The camera focused on participants' face.

#### Non-verbal baseline measures

The automatic detection of facial and head movements was conducted using FaceReader version 8.0, a commercially available software package developed by Noldus Information Technology (2010). FaceReader is an automated program that uses an Active Appearance Model (Cootes, Edwards, & Taylor, 2001) based approach to 3D model the face, identify key points in the face and facial texture, and use Convolutional Neural Networks for facial expression classification to calculate the Action Units (AUs) derived from the FACS, on a frame-by-frame basis. It has been shown to be a reliable measure of automated facial expression analysis (Clark et al., 2020; Dupré, Krumhuber, Küster, & McKeown, 2020). The software estimates activity of AUs from the face. For each frame, software calculates intensity of activation in each AU ranging from 0 (not present) to 1 (maximum). Mean intensity was calculated for each AU across all frames with valid data and used in subsequent analyses. It also captures other forms of non-verbal behavior including head movements along the X, Y, and Z axes. The current study obtained an average of 52 275 (s.d. = 33 813) video frames for each participant. Videos from diagnostic interviews were processed on standard office desktop computers, taking 125% to 150% longer than the original recording. Number of frames was used as a covariate in the analyses to mitigate any potential influence that variations in frame count might exert on the results. In order to quantify head movements, we calculated the frame-by-frame Euclidean distance in head position across the x, y, and z planes following Abbas et al. (for further details see Abbas et al., 2021c). For each participant, mean head movement values were calculated from frame-by-frame Euclidean distance. FaceReader's Action Unit module allows extraction of 20 AU's as described by Ekman et al. (2002, Table 1). FaceReader allowed examination of AU's both laterally and bilaterally. For the purposes of current analyses, only full bilateral values of AUs were used, as lateral AU values were very highly correlated between left and right half.

**Table 1.** Bivariate comparisons of 20 Action Units (AUs) and Head Movements predicting DD onset

	*M*	s.d.	OR	95% CI		*p*
AU 01 – Inner Brow Raiser	0.018	0.030	−0.06	−2.135	0.571	0.256
AU 02 – Outer Brow Raiser	0.0077	0.019	−0.047	−3.156	1.194	0.376
AU 04 – Brow Lowerer*	**0.0115**	**0.0234**	**0.119**	**0.272**	**3.77**	**0.024**
AU 05 – Upper Lid Raiser	0.0107	0.0247	−0.03	−2.295	1.278	0.576
AU 06 – Cheek Raiser	0.0102	0.0163	0.058	−1.088	3.818	0.274
AU 07 – Lid Tightener*	**0.0077**	**0.0153**	**0.105**	**0.056**	**6.092**	**0.046**
AU 09 – Nose Wrinkler	0.0001	0.0007	0.001	−71.383	73.284	0.979
AU 10 – Upper Lip Raiser	0.0058	0.0136	−0.018	−4.059	2.835	0.727
AU 12 – Lip Corner Puller	0.0574	0.0535	0.01	−0.689	0.829	0.857
AU 14 – Dimpler	0	0.0002	0.023	−147.01	228.64	0.669
AU 15 – Lip Corner Depressor	0.0019	0.0082	−0.076	−8.725	1.366	0.152
AU 17 – Chin Raiser	0.0123	0.0242	−0.049	−2.52	0.915	0.359
AU 18 – Lip Pucker	0.0005	0.0018	0.018	−18.345	26.127	0.731
AU 20 – Lip Stretcher	0.0007	0.0021	0.038	−11.762	25.066	0.478
AU 23 – Lip Tightener	0.0003	0.0014	0.004	−27.716	30.046	0.937
Au 24 – Lip Pressor	0.0005	0.0016	−0.056	−39.378	11.957	0.294
AU 25 – Lips Part	0.1007	0.0771	0.062	−0.211	0.843	0.239
AU 26 – Jaw Drop*	**0.015**	**0.0212**	**0.168**	**1.177**	**4.844**	**0.001**
AU 27 – Mouth Stretch	0.0008	0.0026	0.014	−12.907	17.039	0.786
AU 43 – Eyes Closed*	**0.0194**	**0.0347**	**0.104**	**0.009**	**2.381**	**0.048**
Head Movements*	**0.8408**	**0.2914**	**0.147**	**0.06**	**0.342**	**0.005**

*Variables that survived the 5% FDR correction.

#### Self-report, parent-report and physiological baseline measures

To determine the incremental value of non-verbal measures in uniquely forecasting DD, we also examined other baseline predictors of DD. These were selected based on a prior study in which they were found to forecast the first onset of DD in the ADEPT data set (Michelini et al., 2021). These measures included self-report questionnaires tapping depressive symptoms (Inventory of Depression and Anxiety Symptoms General Depression Scales, expanded version; Watson et al., 2012), irritability/hostility (Buss-Perry Aggression Scale; Buss & Perry, 1992), rumination (Response Styles Questionnaire; Nolen-Hoeksema & Morrow, 1991), self-criticism (Depressive Experiences Questionnaire; Blatt, Zohar, Quinlan, Zuroff, & Mongrain, 1995), dependency (Interpersonal Dependency Inventory; Hirschfeld et al., 1977), personality traits (neuroticism/negative affectivity, extraversion/positive affectivity, and conscientiousness) (Big Five Inventory; Soto & John, 2017), relationships with parents and best friend (Network of Relationship Inventory – Relationship Qualities Version; Furman & Buhrmester, 2009), perceived social support (Multidimensional Scale of Perceived Social Support; Zimet, Powell, Farley, Werkman, & Berkoff, 1990), parental warmth (Parental Bonding Instrument; Parker, 1979), and peer victimization (Revised Peer Experiences Questionnaire; De Los Reyes & Prinstein, 2004). History of an anxiety or behavioral disorder in the youth was assessed by KSADS-PL. Parental lifetime history of DSM-IV mood disorder was determined by interviews with participating parents about themselves (Structured Clinical Interview for DSM-IV Axis I Disorders; First, Spitzer, Gibbon, & Williams, 2002) and about the non-participating parents (Family History Screen; Weissman et al., 2000). Parental criticism of the adolescent was assessed through the Five-Minute Speech sample with participating parents (Magaña et al., 1986). See Michelini et al. (2021) for detailed descriptions and psychometric properties of these measures.

## Results

Seventy (19.5%) participants experienced a first-onset DD over 3 years, an average of 14.67 (s.d. = 14.26) months after baseline assessment. Participants with and without a first onset DD did not differ in terms of demographics including baseline age, race, parental education, and household income (online Supplementary Table S1). Hence, these variables were not included in the following analyses.

**Figure 1.**
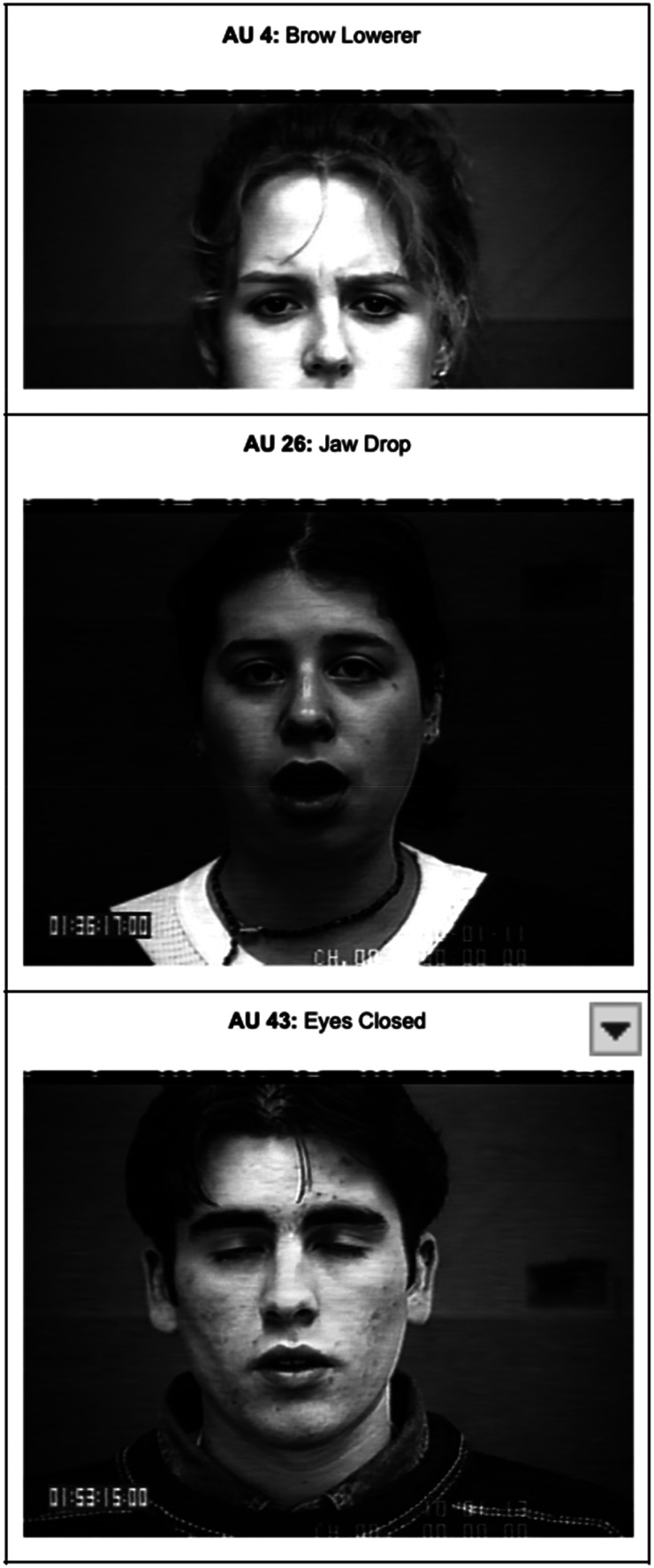
Picture descriptions of AUs that significantly forecast DD at 3-year follow-up.

First, we conducted individual bivariate regressions to assess whether each nonverbal behavior marker is associated with DD onset (see Table 1). Next, logistic regression analysis was conducted to examine non-verbal measures as predictors of DD at 3-year follow-up (Table 2). To avoid bias in the analyses, we used a regression model in which the data guided the determination of the key predictors in the model. This was implemented with a forward regression approach in which DD at 3-year follow-up was the dependent variable. In the forward regression, 3 AUs and head movements were significant in forecasting DD at 3-year follow-up: mean brow lowerer (AU4), jaw drop (AU26), eyes closed (AU43) and head movement (Table 2; Fig. 1). Among these significant predictors, head movements, AU4, and 26 survived %5 FDR correction, therefore were included in the further analyses, while AU43 was excluded. As a follow-up analysis, the number of analyzable valid frames from each participant's video was included in the analysis as a covariate, however it did not impact the significant findings.

**Table 2.** Forward-Entry Logistic Regression predicting DD at 3-year follow-up with nonverbal behaviors

	Beta	s.e.	Wald	df	*p*	OR	95% CI	
							Lower	Upper
AU 4 – Brow Lowerer	0.306	0.117	6.862	1	0.009	1.357	1.08	1.706
AU 26 – Jaw Drop	0.394	0.122	10.46	1	0.001	1.483	1.168	1.884
AU 43 – Eyes Closed	0.27	0.118	5.24	1	0.022	1.309	1.04	1.649
Head Movements	0.396	0.132	8.996	1	0.003	1.485	1.147	1.923

The entry of independent variables was set at 0.05 and variable removal was set at 0.10. Independent variables in the model included mean values of 20 AUs and head movements. All continuous variables were standardized.

**Figure 2.**
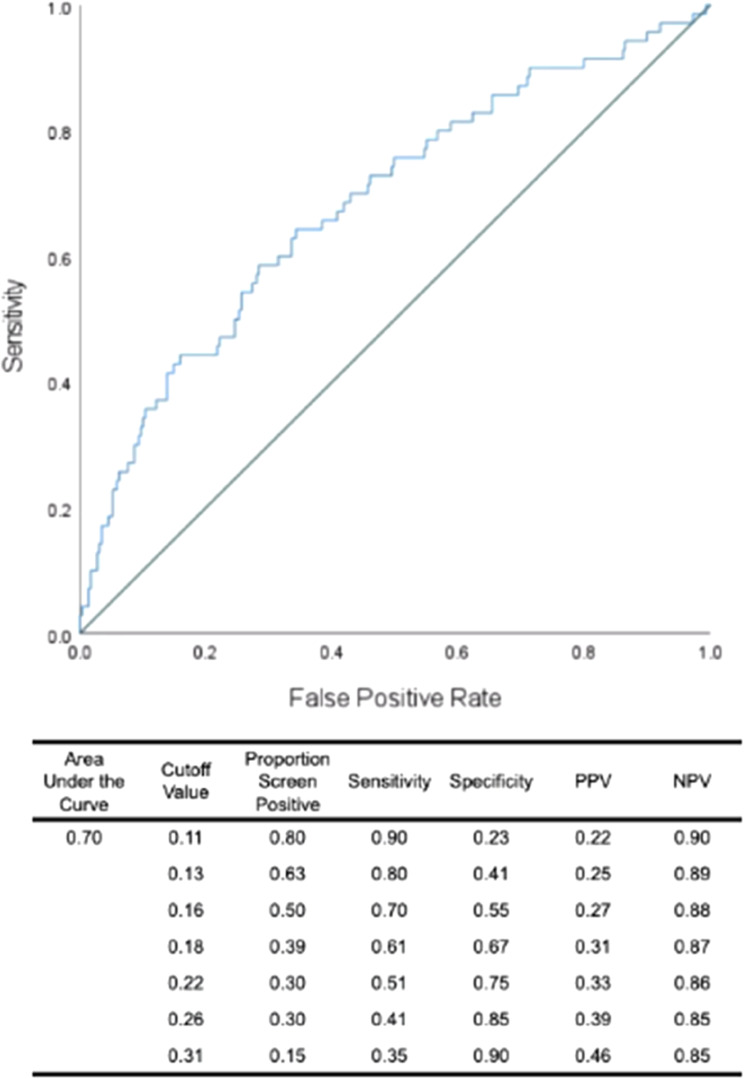
ROC curve for the logistic regression predicting DD at 3-year follow-up with nonverbal behaviors.

Receiver operating characteristic (ROC) curve analyses were performed to calculate area under the curve (AUC), sensitivity, specificity, positive and negative predictive value for probability of DD onset estimated by the model (i.e. a weighted composite of the four behavioral predictors) (Fig. 2). The AUC was 0.70, suggesting that nonverbal head movements and facial AUs have moderate to low accuracy in discriminating between individuals who will and will not develop a first onset of a DD (Streiner & Cairney, 2007).

**Figure 3.**
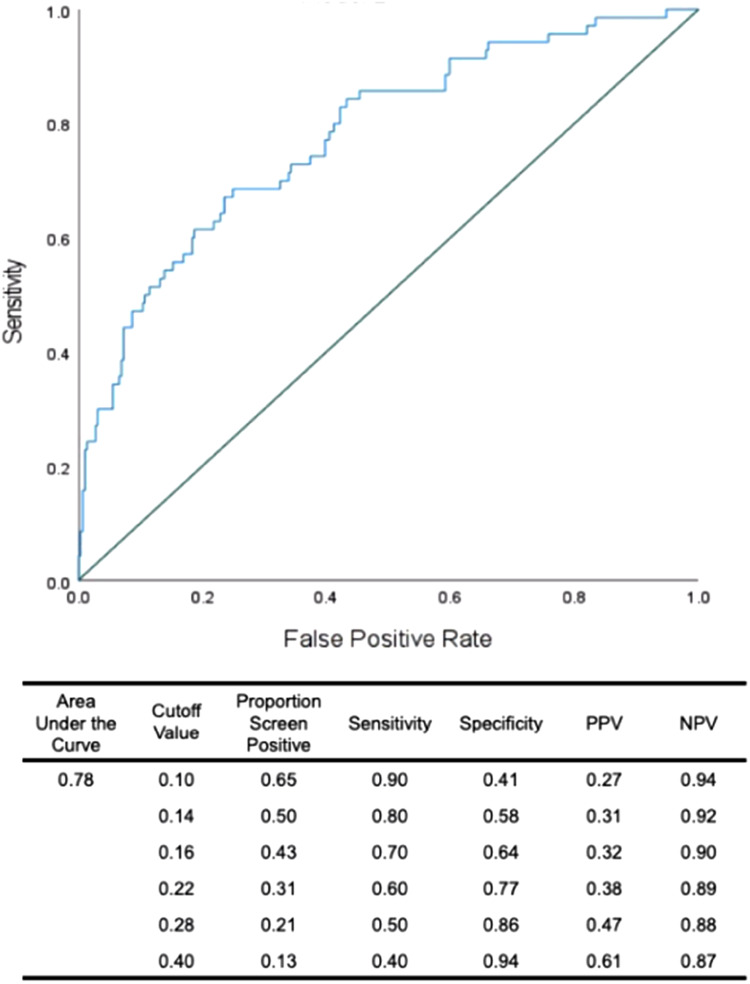
ROC curve for the logistic regression demonstrating incremental validity of nonverbal behaviors along with previously established risk markers of DD at 3-year follow-up.

Next, we examined whether non-verbal movements have incremental value in forecasting future depression beyond previously established measures. We confirmed that 13 of the 16 traditional risk factors were selected in the present sample (see online Supplementary Table S2), and were included in a second forward entry binary logistic regression with mean head movements and significant AUs. Head movement, brow lowerer (AU4), and jaw drop (AU26) emerged as unique predictors of DD at 3-year follow-up, along with baseline IDAS and RSQ scores (Table 3). ROC analysis demonstrated a moderate accuracy with AUC value of 0.78 (Fig. 3). With lower cutoff, the risk composite showed high sensitivity (0.90), although with the downside of selecting 65% of the sample; thus, the risk composite can be used to screen out adolescents whose risk of DD onset is very low.

**Table 3.** Logistic Regression of head movements, significant action units (AUs) and baseline self- & parent-reported and biological measures predicting DD at 3-year follow-up

	Beta	s.e.	Wald	df	*p*	OR	95% CI	
							Lower	Upper
Depressive Symptoms	0.557	0.187	8.905	1	0.003	1.746	1.211	2.517
Rumination	0.443	0.178	6.201	1	0.013	1.557	1.099	2.206
Head Movements	0.342	0.141	5.86	1	0.015	1.408	1.067	1.858
AU 04 – Brow Lowerer	0.387	0.133	8.517	1	0.004	1.473	1.136	1.911
AU 26 – Jaw Drop	0.47	0.151	9.661	1	0.002	1.6	1.19	2.152

In this model, depression diagnosis at 3-year follow-up was again used as the dependent variable, and the same model parameters were used.

**Figure 4.**
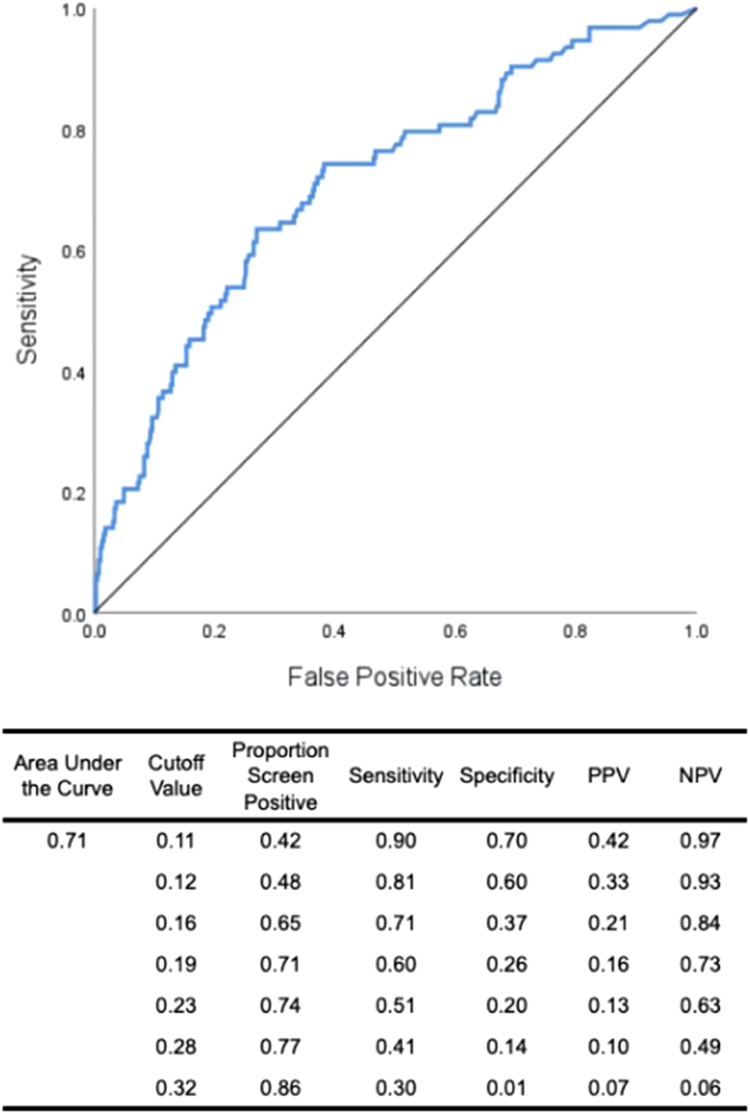
ROC curve for the logistic regression including IDAS-depression and RSQ predicting DD status at 3-year follow-up.

Finally, we have created a logistic regression model for the significant psychosocial predictors (IDAS Depression and RSQ) predicting DD status at 3-year follow-up (see Fig. 4) to demonstrate the predictive value of these variables without nonverbal behavior markers.

## Discussion

The clinical and public burden of DD can be mitigated by early prevention efforts that are shown to reduce rates and alleviate the course of the illness (Harrington & Clark, 1998; Ormel, Cuijpers, Jorm, & Schoevers, 2019). While studies show that prevention efforts for adolescents who are at high risk for developing DDs is effective, identification of these individuals remains a major challenge for the field (Kieling et al., 2019). The present study aimed to explore whether nonverbal behaviors, captured by digital assessment tools during a clinical interview in adolescence, may forecast the first onset of DD in 3 years. Our findings demonstrated that nonverbal behaviors, including greater head movement, AU4 (brow lowerer), AU26 (jaw drop), and AU43 (eyes closed) show promise in indexing future DD risk.

Critically, most of these digital measures showed incremental value in predicting depression beyond the previously established predictors of first onset depression in this sample (Michelini et al., 2021). Increased movements of the head as well as AU4 indexing brow lowering, and AU26 indexing jaw dropping were the non-verbal measures that entered the model predicting DD at 3-year follow-up, while many other clinical and psychosocial measures, such as baseline family psychiatric history, personality traits, and peer victimization, did not. Importantly, these non-verbal measures accounted for unique variance over and above baseline self-reported depression score and rumination, which are some of the strongest predictors of future depression (Michelini et al., 2021). Our non-verbal measures are advantageous in providing an objective, scalable, non-invasive, and cost-effective approach for early risk assessment of DD. The findings from the present study can address various clinical needs depending on the cutoff values applied. For example, when cutoffs that enhance sensitivity (even with lower specificity) are used, these digital assessments can effectively support widespread screening initiatives. In contrast, cutoffs that prioritize specificity (despite low sensitivity) make these assessments more suitable for confirmation following initial screenings or for use in specialized settings where the illness is more prevalent (Baldessarini, Finklestein, & Arana, 1983).

This is the first study on the digital assessment of nonverbal behaviors that has been conducted with a longitudinal community sample of adolescents. The literature so far has primarily used cross-sectional designs, attempting to detect the presence of DD within a sample, with a few studies examining prediction of the course of the illness over a few months (Dibeklioğlu et al., 2018; Kacem et al., 2018). The present study breaks new ground in forecasting the future first onset of DD during a critical developmental window, with a rich variety of clinical predictors along with nonverbal behavior.

The specific nonverbal behaviors that emerged as having predictive ability in the current study complement prior research. Our results showing that greater movement in AU4 (brow lowerer) predicts DD is in line with research showing that heightened intensity in this AU indexes the underlying activity of the corrugator muscle, which has been shown to be closely associated with depression (Kadison, Ragsdale, Mitchell, Cassisi, & Bedwell, 2015). Moreover, early EMG studies found that amplified corrugator muscle activity, which contributes to a frowning facial expression, may predict prognosis, as well as diagnosis of DD (Greden et al., 1984; Schwartz et al., 1978). On the other hand, although the examination of masseter muscle activity underlying AU26 (jaw dropping) is limited in clinical science literature, the dental literature extensively reports associations between higher self-reported depressive symptoms and EMG measured masseter muscle activity, particularly in women (Gonzalez, Nickel, Scott, Liu, & Iwasaki, 2018), and these findings are associated with jaw related temporomandibular disorders (Khawaja et al., 2015).

On the other hand, head movements have recently received considerable attention in studies that use digital assessment tools to identify current DD. However, our results depart from prior studies that predominantly linked DD with reduced head movement (Alghowinem et al., 2013; Joshi et al., 2013). Over time, head movement in patient videos increased as depression symptoms ameliorated in the laboratory (Girard et al., 2014) and remote assessments via smartphone-based video analyses (Abbas et al., 2021a). Several factors could explain the observed increase rather than decrease in head movement activity in the present study. Notably, previous research exclusively involved currently depressed adult participants during non-verbal data acquisition. In contrast, the present sample involves *healthy* adolescent participants who might be *at risk* for DD. In such a sample, the pattern of relationship between motor movement characteristics and DD might manifest differently.

Depression is characterized by both observable slowing of (psychomotor retardation) as well as increased (agitation) psychomotor movement (American Psychiatric Association, 2013; Sobin, 1997). Specifically, psychomotor agitation is defined as ‘restless physical activity arising from mental disturbance’ (APA Dictionary of Psychology, n.d.). The increased average head movements that are measured in 3D space is a potential method to quantify psychomotor agitation. In adolescence, self-reported psychomotor agitation has been demonstrated to couple with family loading of risk for depression, forecast future depression symptoms in 1-year (Damme et al., 2021), and index a transdiagnostic risk marker for both DD and psychotic-like experiences (Damme et al., 2022). Hence, it is possible that greater overall head movements reflect psychomotor agitation which might be a prominent marker of risk for DD that is unique to adolescence.

Second, phenotypic expressions of DD are highly heterogeneous and nuanced. Both psychomotor agitation and retardation may represent different subtypes (Leventhal et al., 2008; Schrijvers, Hulstijn, & Sabbe, 2008). Furthermore, there is weak evidence regarding gender differences in psychomotor movements of DD. Some early studies suggest potential sex-related differences in psychomotor abnormalities where some studies report more pronounced psychomotor agitation in females than males (Avery & Silverman, 1984; Sobin, 1997; Winokur, Morrison, Clancy, & Crowe, 1973), while others report mixed or null results (Khan, Gardner, Prescott, & Kendler, 2002; Kornstein et al., 2000). However, questions regarding nonverbal behavior were not pursued extensively in more recent decades and psychomotor agitation of gross movements remained largely unexamined (Schrijvers et al., 2008).

Furthermore, it is crucial to emphasize that in the current study, the digital assessment of nonverbal behaviors was conducted within the context of clinical diagnostic interviews. Consequently, the heightened head movement observed in participants who subsequently developed a DD might be a proxy of the significant distressing issues they shared during the interview, even though they had not yet experienced a DD at the time. In contrast, well-adjusted participants might have fewer issues to report, resulting in a more stable psychomotor movement during the interview.

Overall, nonverbal psychomotor behavior has long been viewed as a potential diagnostic and risk marker in clinical science, however research has been limited and constrained due to flaws with assessment methods (Schrijvers et al., 2008; Sobin, 1997). Digital phenotyping currently offers an exciting and novel approach for objective, scalable, cost-effective assessment of nonverbal behaviors to address the essential need for early identification of DD vulnerability. Furthermore, nonverbal behavior can be indicative of underlying mechanisms of depression (Girard & Cohn, 2015), as it is not limited to muscle contractions but involves perceptual processes and cognitive-control mechanisms that underlie the muscle activity (Schrijvers et al., 2008). In fact, electrical stimulation of facial musculature has been recently proposed as a potential intervention method for DD. As nonverbal psychomotor behavior may reflect underlying pathophysiology, changing facial muscle activity may have an impact on affect, in line with the facial feedback hypothesis (Demchenko et al., 2023).

There are a number of limitations of current study. The sample was predominantly white, mirroring the composition of the study sample and representing the demographic picture of Suffolk County, New York, and limited to female adolescents. To enhance the robustness of the findings and broaden applicability, replication of the study with diverse demographic profiles, including varying racial, ethnic, and cultural groups, is imperative (Barrett, Adolphs, Marsella, Martinez, & Pollak, 2019). On the other hand, there is recent evidence demonstrating a relatively high level of universality of nonverbal facial movements. By utilizing machine learning methods, evidence suggests 70% consistency of facial expressions in similar contexts, such as wedding as sports games, across 144 countries in 12 different world regions (Cowen et al., 2021). Future research should examine the applicability of findings related to nonverbal behaviors and DDs to broader and more diverse contexts, both encompassing demographic and clinical diversity. In addition to replication, it is crucial to assess the extent to which current facial recognition technology permits generalization to diverse populations and real-world applicability. Specifically, for the computer-vision-based facial recognition technology, there are concerns regarding the generalizability and equity of the methods (Buolamwini & Gebru, 2018), underscoring the need for carefully considering technology's potential biases. Algorithms rapidly adopt and reflect societal biases, including racism and sexism, which are now well-documented in current facial recognition technology. The high accuracy of facial identification is not universal; it is primarily effective for European white and male facial features. In contrast, error rates for darker-skinned females can reach up to 34% (Buolamwini & Gebru, 2018; Phillips, O'Toole, Jiang, Narvekar, & Ayadd, 2011). This issue has not been documented for facial emotion recognition algorithms. In particular, FaceReader was trained on a set of faces that included many African and Asian individuals (Spink, Barski, Brouwer, Riedel, & Sil, 2022). However, racial biases may be presented in emotion recognition models, even if not detected or will emerge in the future. Consequently, deploying these technologies in real-world applications without extensive and meticulously conducted research and considerations can exacerbate existing inequalities in the mental health system (Maura & Weisman de Mamani, 2017), by potentially leading to inaccurate assessments, misdiagnoses, or overlooked symptoms in underrepresented populations.

Moreover, researchers and clinicians should exercise caution in employing digital methods in both research and real-world applications. While these methods hold great promise, it is crucial to acknowledge that the validation and regulation of digital measurement tools present a spectrum of ethical and privacy-related concerns. One primary issue is the potential for invasions of privacy, as facial recognition involves the collection and storage of sensitive biometric data, which can be prone to misuse or unauthorized access. There are also concerns about consent, as individuals may not fully understand how their data will be used or the implications of its collection. Finally, protection and regulations against mishandling and exposure of facial recognition data holds significant implications for further stigmatization and discrimination due to mental health conditions. Ensuring robust safeguards, transparency, and patient consent is essential prior to the application of facial recognition technology in mental health care. These concerns should be scrutinized closely in future research to ensure responsible and ethical use of such tools.

Overall, nonverbal psychomotor behavior has long been viewed as a potential diagnostic and risk marker in clinical science, however research has been limited and constrained due to flaws with assessment methods (Schrijvers et al., 2008; Sobin, 1997). For example, it can be integrated into the routine preventive care visits at a pediatrician's office, contributing to a broader evaluation that includes current symptoms of depression, anxiety, and rumination. Effect sizes that we observed for nonverbal behavior were too small to use them in isolation; these behavioral markers show potential to enhance prediction of future DD as part of a larger panel of risk factors. Nevertheless, additional research is essential to enhance precision of these markers. The current study is unique in presenting a multimodal assessment of longitudinal risk factors for DD in a large sample of adolescent girls. It is important to reiterate that most research studies in the literature that use digital methods to examine nonverbal behavior only predict concurrent depressive symptoms and the few longitudinal studies only follow participants for a limited duration. Our study stands out as following a non-depressed group of adolescents over 3 years, which covers a large span of the significant developmental period. By providing a comparison to other, better established, clinical and psychosocial risk markers, our study fills an important gap in the literature (Cohn et al., 2018). Thereby, it underscores the capacity of facial recognition as a promising avenue for future research. With further research support, it can be applied to diverse clinical settings with minimal effort, resources and training, to quantify nonverbal movements objectively, so that clinicians can access an efficient, easy, cost-effective tool for risk assessment that would aid in prevention and intervention of future DD. Future research should replicate these findings extensively with diverse demographic profiles and phenotypic expressions of DD, in combination with multimodal assessment of DD signs and risk.

## Supporting information

Ozturk et al. supplementary material Ozturk et al. supplementary material

## Supplementary material

The supplementary material for this article can be found at https://doi.org/10.1017/S0033291724002010.
