# Supplementary material for: Digital assessment of nonverbal behaviors forecasts first onset of depression
Source: Psychol Med. 2024 Oct 4;54(12):3507–18. doi: 10.1017/S0033291724002010 (PMC11496224; doi:10.1017/S0033291724002010)

**Supplementary Methods**

**Table 1.** Demographic details of the sample

**
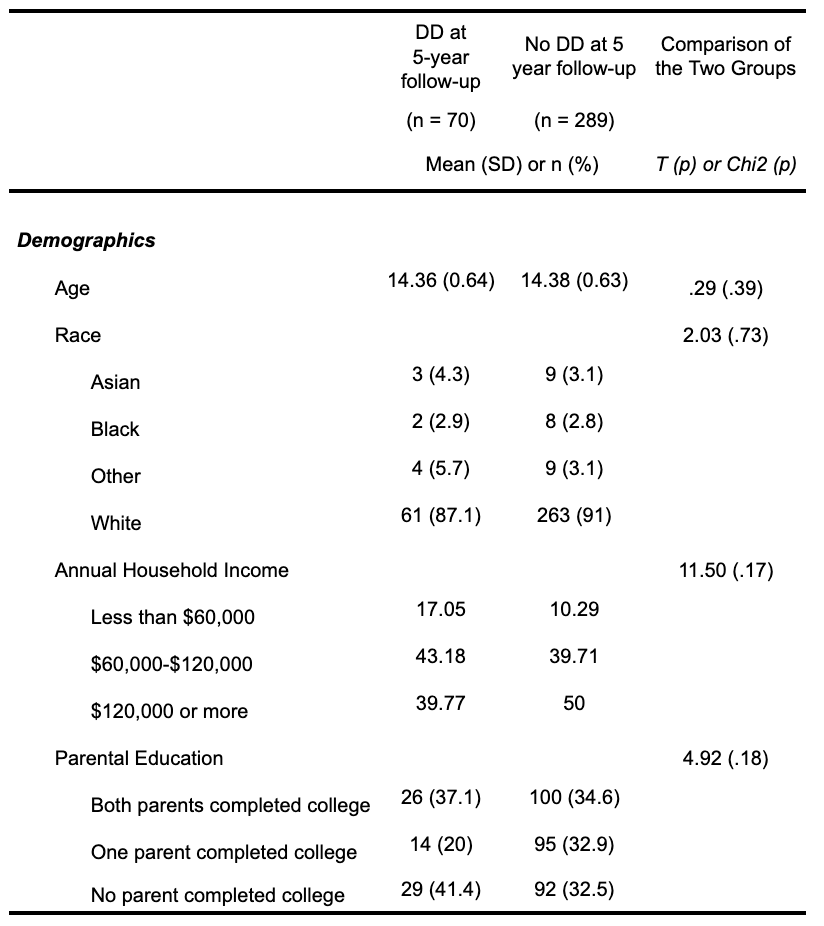
**

**Supplementary Results**

**Table 2.** Bivariate associations between baseline predictor measures established by Michelini et al. (2021) and DD at 3-year follow-up


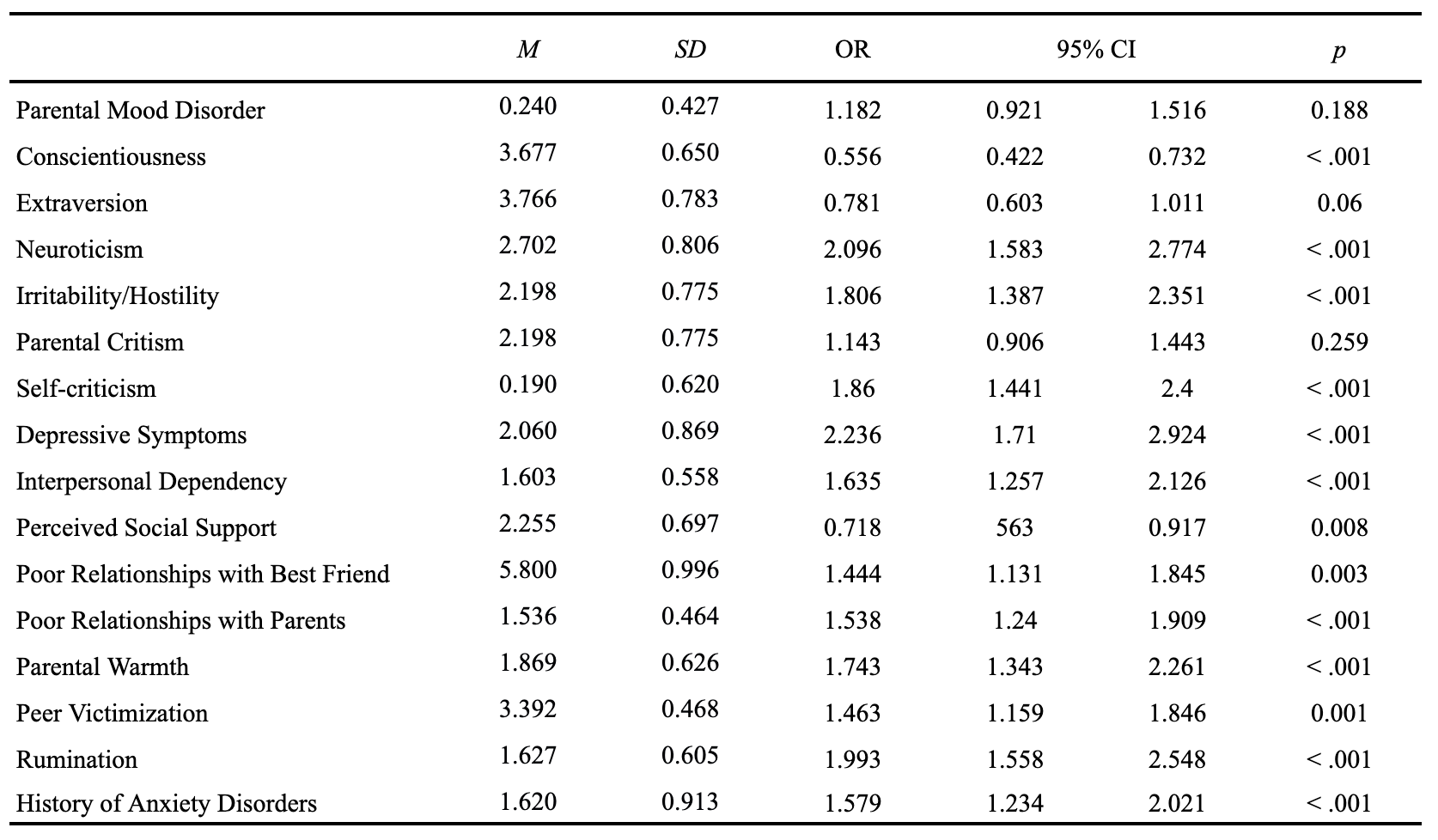

Supplement: Ozturk et al. supplementary material [file S0033291724002010sup001.docx]
